# The prevalence and systemic risk factors of diabetic macular edema: a cross-sectional study from Turkey

**DOI:** 10.1186/s12886-018-0753-y

**Published:** 2018-04-12

**Authors:** Durgul Acan, Mehmet Calan, Duygu Er, Tugba Arkan, Nilufer Kocak, Firat Bayraktar, Suleyman Kaynak

**Affiliations:** 1 Department of Ophthalmology, Dokuz Eylul University School of Medicine, Izmir, Turkey; 2 Department of Endocrinology and Metabolism, Dokuz Eylul University School of Medicine, Izmir, Turkey

**Keywords:** Diabetic macular edema, Optical coherence tomography, Prevalence

## Abstract

**Background:**

The aim of this study was to evaluate the prevalence of diabetic macular edema (DME) utilizing optical coherence tomography (OCT), and to clarify the effects of the systemic findings and risk factors on the development of DME.

**Methods:**

This cross-sectional study was conducted in the departments of ophthalmology and endocrinology at the Dokuz Eylul University School of Medicine in Izmir, Turkey. The demographics, type and duration of diabetes mellitus, treatment modality, smoking and alcohol consumption habits, as well as the systemic blood pressure, renal functional tests, hemoglobulin A1c level, serum lipid profile, and 24-h urine albumin level were noted and statistically analyzed. The relationships between the systemic findings and DME were studied.

**Results:**

Four-hundred and thirteen eyes of 413 diabetic patients who were examined between January 2011 and July 2012 were enrolled in this study. The prevalence of DME was 15.3% among the patients. The males exhibited DME significantly more frequently than the females (*p* = 0.031), and the duration of diabetes was significantly longer in those patients with DME (*p* < 0.001). Those patients without DME frequently used antihyperlipidemic drugs and had a higher level of high density lipoprotein cholesterol (*p* = 0.040 and *p* = 0.046, respectively). The patient’s alcohol consumption, nephropathy, neuropathy, previous cataract surgery, severity of diabetic retinopathy, and insulin usage were statistically significant factors with regard to the DME prevalence.

**Conclusions:**

This study demonstrated the prevalence of DME in Turkey by utilizing OCT. The development of DME can be avoided or limited and the response to treatment may be improved by the regulation of the DME risk factors.

## Background

Since 1980, the adult population living with diabetes has increased four-fold to approximately 422 million according to the most recent World Health Organization’s *Global Report on Diabetes*. This sharp rise can be attributed to overweight and obesity, which have resulted in an increase in type 2 diabetes [1]. The prevalence of diabetes in Turkey has recently been reported as 13.2% [2].

The most common reason for vision loss in diabetic patients is diabetic macular edema (DME). Unfortunately, the absolute prevalence of DME may be increasing due to the overall increase in the prevalence of diabetes in industrialized nations [3]. Population-based studies have reported the prevalence of DME in type 1 diabetic patients as 4.2–7.9%, while the rate for type 2 diabetes patients ranges from 1.4–12.8% [4–27]. In a Cochrane review of the DME prevalence evaluated using optical coherence tomography (OCT), the prevalence rates covered a wide range (19%–65%) [28].

In recent years, the use of OCT has become more widespread for the objective measurement of retinal thickness and the other elements of macular edema [29–31]. The Diabetic Retinopathy Clinical Research network (DRCR.net) has adopted standard OCT DME assessments in multicenter studies of diabetic retinopathy (DR). Since this assessment is quantitative with the use of OCT, rather than qualitative when applying photography or biomicroscopy, this is considered to be a significant advantage.

The epidemiology and disease burden have not yet been fully elucidated, and there is limited information on the current state of DME in Turkey. Therefore, the aim of this study was to evaluate the prevalence, demographic characteristics of the patients, and systemic associations of DME utilizing OCT in Izmir, Turkey.

## Methods

This cross-sectional study was conducted in the departments of ophthalmology and endocrinology at the Dokuz Eylul University School of Medicine in Izmir. A total of 413 eyes of 413 diabetic patients who were followed up in the clinics between January 2011 and July 2012 were enrolled. The demographic data, diabetes type, diabetic age, treatment modality, smoking and alcohol consumption habits, as well as the systemic blood pressure, renal functional test results, hemoglobulin A1c (HbA1c) level, serum lipid profile, 24-h urine albumin level, and the existence of neuropathy were noted and statistically analyzed. The ophthalmological evaluation of each participant included the best corrected visual acuity (BCVA), slit-lamp biomicroscopy, intraocular pressure (IOP) measurement, and dilated fundoscopy. Fluorescein angiography and a central macular thickness (CMT) analysis with OCT were also performed. The relationships between the systemic findings and the prevalence of DME were studied.

Those patients ≥18 years old with type 1 or 2 diabetes diagnosed by an endocrinologist at the Dokuz Eylul University Hospital Endocrinology Clinic between January 2011 and July 2012, who were then referred to the Ophthalmology Department Retina Unit for DME and DR screenings, were included in this study.

The exclusion criteria were as follows: eyes with an ocular abnormality other than DME (vitreomacular traction, epiretinal membrane, etc.) and media opacities interfering with the reliability of OCT imaging (dense cataract, uveitis, etc.), and those patients with insufficient data for the study protocol.

### Ophthalmological examination

The BCVA was evaluated using the Bailey-Lovie chart after correcting for refractive errors. An anterior segment examination was conducted using slit-lamp biomicroscopy and dilated fundoscopy. The IOP was obtained with a Goldmann applanation tonometer, and Heidelberg retinal angiography (HRA) and OCT were performed using the Spectralis HRA-OCT II (Heidelberg, Germany). After obtaining a fixation point for the patient, 6 OCT shots were lined up with the radial line scan and each other at an angle of 30°. The eyes were evaluated for clinically significant macular edema (CSME) as defined by the Early Treatment Diabetic Retinopathy Study (ETDRS) and with a central macular thickness (CMT) (mean thickness at the point of the intersection of 6 radial scans) via OCT ≥ 250 μm attributable to DME [32].

### Statistical analysis

The data from all of the subjects who fulfilled the inclusion/exclusion criteria were analyzed using SPSS 16.0 software. For the descriptive analysis, the mean, standard deviation, and percentage were used. The chi-squared test, Fisher’s exact test, and t-test were applied for the univariate analysis. A *p* value < 0.05 was considered to be statistically significant.

## Results

Of the 425 patients who met the study criteria, 413 were included for evaluation. DME was detected in 15.3% (63) of the patients and DR was determined in 32% (132) of the patients. Moreover, DME was found in 14.8% (4) of the patients with type 1 diabetes and in 15.3% (59) of the patients with type 2 diabetes (*p* = 0.604). Of the 63 DME patients, 15 received previous focal/grid laser treatments, 8 received previous intravitreal anti-vascular endothelial growth factor (VEGF) or steroid treatments, and 5 received previous combined focal/grid laser and anti-VEGF/steroid treatments. In addition, 9 patients without DME received previous focal/grid treatments and one patient underwent a vitrectomy.

The demographic and laboratory characteristics of the patients are summarized in Table 1. DME was significantly more prevalent in the males than the females (*p* = 0.031), and the male subjects had higher HbA1c levels than the female subjects (8.30 ± 2.25% and 7.89 ± 2.13%, respectively) (*p* = 0.054). Although there was no direct statistical correlation between the HbA1c levels and DME, a significant increase in the frequency of DME was observed particularly in those subjects with HbA1c values of 7.0% or more (*p* = 0.037). While the type of diabetes did not have an effect on DME, the duration of diabetes was significantly longer in the DME patients, particularly in those diagnosed between 10 and 20 years previously (*p* < 0.001). Those patients without DME were determined to have a significantly higher rate of antihyperlipidemic drug usage and a higher level of high density lipoprotein cholesterol (HDL-C) (*p* = 0.040 and *p* = 0.046, respectively). The mean serum creatinine levels in those patients with and without DME were 1.13 ± 0.81 mg/dL and 0.87 ± 0.63 mg/dL, respectively, and this difference was statistically significant (*p* = 0.021).

**Table 1:** Comparison of the demographic and laboratory characteristics of the patients with and without DME

Characteristics	Patients with DME (*n* = 63, 15.3%)	Patients without DME (*n* = 350, 84.7%)	*P* value
Age (years)	58.86 ± 11.27	56.03 ± 11.95	0.082
Gender (female/male)	26/37	196/154	0.031*
BMI (kg/m^2^)	29.25 ± 5.78	29.46 ± 5.80	0.797
Type of diabetes (1/2)	4/59	23/327	0.604
Duration of diabetes (years)	16.77 ± 8.16	7.64 ± 7.12	< 0.001*
DR (n)			
Mild-moderate DR	18 (28.6%)	52 (14.9%)	< 0.001*
Severe-very severe	10 (15.9%)	6 (1.7%)	
PDR	35 (55.5%)	11 (3.1%)	
Smoking (*n* = 97, 23.5%)	12 (19%)	85 (24.2%)	0.367
Alcohol (*n* = 10, 2.4%)	5 (7.9%)	5 (1.4%)	0.010*
Hypertension (*n* = 242, 58.5%)	42 (66.6%)	200 (57.1%)	0.158
Systolic blood pressure (mmHg)	130.37 ± 20.15	128.56 ± 17.86	0.469
Diastolic blood pressure (mmHg)	79.25 ± 8.87	79.05 ± 9.91	0.881
Anti-hyperlipidemic drug usage (*n* = 109, 26.4%)	10 (15.9%)	99 (28.3%)	0.040*
CVD (*n* = 78, 18.9%)	16 (25.4%)	62 (17.7%)	0.152
Peripheral neuropathy (*n* = 209, 50.6%)	42 (66.6%)	167 (47.7%)	0.006*
Nephropathy (*n* = 99, 24.0%)	28 (44.4%)	71(20.3%)	< 0.001*
Normoalbuminuria (*n* = 314, 76%)	35 (55.5%)	279 (79.7%)	< 0.001*
Microalbuminuria (*n* = 69, 16.7%)	20 (31.7%)	49 (14%)	
Macroalbuminuria (*n* = 30, 7.3%)	8 (12.7%)	22 (6.2%)	
HbA1c (%)	8.39 ± 1.97	8.02 ± 2.23	0.226
FBG (mg/dL)	164.50 ± 57.86	157.50 ± 65.84	0.523
Creatinine (mg/dL)	1.13 ± 0.81	0.87 ± 0.63	0.021*
GFR (mL/min/1.73 m^2^)	76.21 ± 28.82	88.25 ± 22.35	0.002*
Total cholesterol (mg/dL)	187.95 ± 41.75	194.00 ± 50.72	0.372
LDL-C (mg/dL)	114.44 ± 33.76	117.85 ± 36.72	0.494
HDL-C (mg/dL)	40.19 ± 11.87	43.45 ± 11.92	0.046*
Triglyceride (mg/dL)	165.24 ± 86.87	158.52 ± 113.69	0.656

Results are given as the mean ± SD. A *p* value of < 0.05 was considered to be significant (*). *BMI* body mass index, *CVD* cardiovascular disease, *CMT* central macular thickness, *DR* diabetic retinopathy, *HbA1* hemoglobin A1c, *GFR* glomerular filtration rate, *FBG* fasting blood glucose, *HDL-C* high density lipoprotein cholesterol, *LDL-C* low density lipoprotein cholesterol, *PDR* proliferative diabetic retinopathy

In the comparison of the normoalbuminuric, microalbuminuric, and macroalbuminuric patients in terms of the DME frequency, a statistically significant difference was seen between the 3 groups (*p* < 0.001). While 11.0% of the patients without nephropathy had DME, 29.0% of patients with microalbuminuria and 26.7% of the patients with macroalbuminuria had DME (*p* < 0.001). Peripheral neuropathy was also significantly frequent in those patients with DME (*p* = 0.006). The mean BCVAs of the eyes with and without DME were 0.55 ± 0.59 logMAR and 0.04 ± 0.10 logMAR, respectively (*p* < 0.001). The mean IOPs of the eyes with and without DME were 14.91 ± 2.45 mmHg and 15.12 ± 2.64 mmHg, respectively, and no statistical difference was seen (*p* = 0.562).

The prevalence of DME was 28.6% in those patients with mild to moderate non-proliferative diabetic retinopathy (NPDR) and 72.6% in those patients with severe NPDR to proliferative diabetic retinopathy (PDR) (*p* < 0.001). The DME prevalences in the phakic and pseudophakic eyes were 12.9% (49) and 43.7% (14), respectively (*p* < 0.001). Assuming the possible effects of cataract surgery on DME and evaluating only the phakic patients showed that the duration of diabetes, nephropathy, neuropathy, and antihyperlipidemic drug use significantly affected the DME in similar ways (*p* < 0.001, *p* = 0.020, *p* = 0.012, and *p* = 0.038, respectively). However, in the phakic patients, the gender, creatinine level, and HDL-C level did not have statistically significant effects on the DME (*p* = 0.610, *p* = 0.227, and *p* = 0.233, respectively).

## Discussion

There is a known increasing worldwide prevalence of DME. Correspondingly, an increase in diabetes-related complications is expected with the increase in diabetes mellitus cases in Turkey. In one study from Turkey, the prevalence of DME was found to be 14.2% in the pre-OCT era [33]. Most studies have used non-stereoscopic fundus photography; therefore, the accuracy of the DME assessment is in doubt. The use of stereoscopic slit-lamp biomicroscopy alone may also lead to both the underdiagnosis and overdiagnosis of DME. Macular edema was defined using the CSME criteria in approximately one-half of the previous studies, and thus, only covered the more severe DME spectrum. The clinical use of OCT has enabled the detection of DME that was previously overlooked in a stereoscopic fundus examination. When compared to a clinical examination, the OCT detection and assessment of DME is more objective and reproducible, ensuring greater uniformity in the interventions applied and the treatment outcomes when compared to the pre-OCT era [34, 35]. According to DRCR.net, for DME trial inclusion and retreatment eligibility, the central subfield mean thickness on a Stratus OCT must be ≥250 μm. The current study used the Spectralis HRA-OCT II, which produces high resolution histological macular images, and the prevalence of DME was found to be 15.3%. This ratio was higher than the prevalence in a previous study conducted in 2006, and thus supports the sensitivity of the OCT.

The DME prevalence is related to the disease duration. In the present study, the prevalence of DME was 2.8% within 5 years of the diabetes diagnosis and 22.0% 5 years after the diagnosis (*p* < 0.001). After 10 years, the prevalence rose prominently. In a study by Aiello et al. [36], the prevalence was 5% within the first 5 years after the diagnosis and 15% at 15 years.

The males in this study exhibited DME more frequently than the females, and the odds ratio (OR) for the males was 1.811 (95% CI: 1.051 < OR < 3.121) (*p* = 0.031). In addition, the HbA1c levels were significantly higher in the males than the females; therefore, and it can be suggested that not only gender, but also worse diabetic control in male patients can indicate a higher prevalence of DME. The HbA1c level in the patients with DME (8.39 ± 1.97%) was slightly higher than that in the patients without DME (8.02 ± 2.23%), but this difference was not statistically significant (*p* = 0.226). The prevalences of DME in those patients with HbA1c levels < 7.0% and ≥ 7.0% were 10.62% and 18.18%, respectively (*p* = 0.037). In the Diabetes Control and Complications Trial (DCCT), it was shown that the strict control of blood glucose in type 1 diabetes patients led to a 29% decrease in the cumulative incidence of macular edema at the 9-year follow-up, and halved the application of focal laser treatment for DME [37, 38]. Even if there is a deterioration in control later in life, the effects of improved glycemic control sustained over many years have been shown to persist. In the Epidemiology of Diabetes Interventions and Complications (EDIC) study, which was an extension of the DCCT in which the level of glycemic control of the former intensive and conventional control groups converged, it was reported that the former intensive control group continued to fare better than the former conventional control group. Four years after the end of the DCCT, the CSME incidence was 2% in the former intensive control group, compared to the 8% rate in the former conventional control group (*p* < 0.001) [39].

In the UK Prospective Diabetes Study, an analogous, randomized clinical trial of type 2 diabetes patients, it was reported that strict blood glucose control resulted in a 29% reduction in laser treatment in a follow-up period of 10 years; of the laser treatments required, 78% were for DME [40]. In the current study, the prevalence of DME was conspicuously higher in the insulin-taking patients (*p* < 0.000). In previous studies, taking insulin has been reported to trigger the development of DME in the acute period. In this period, the hypoxia-inducible factor connects to the VEGF promotor region, and the VEGF transcription increases. Subsequently, the blood-retina barrier breaks down and permeability increases with the activation of protein kinase C. In the chronic period, insulin shows anti-inflammatory and anti-apoptotic effects and reduces oxidative stress [41]. The high prevalence of DME in the insulin-taking patients in the present study may be the result of the poor glycemic control in these patients.

The UK Prospective Diabetes Study also reported that the mean systolic blood pressure was reduced by 10 mmHg and the diastolic blood pressure was reduced by 5 mmHg in a median follow-up period of 8.4 years, which resulted in a 35% decrease in the retinal laser treatments, 78% of which were for DME [42]. In addition, the Wisconsin Epidemiologic Study of Diabetic Retinopathy determined that systemic hypertension increased the prevalence of DME 3-fold. It has been suggested that not only is hypertension a risk factor for macular edema development, but the treatment may have important benefits in patients with uncontrolled hypertension [43]. In the current study, the prevalences of DME in those patients with and without systemic hypertension were 17.4% and 12.3%, respectively (*p* = 0.158). In addition, there was no statistically significant difference with respect to the systolic and diastolic blood pressure levels between those patients with and without DME. However, anti-hypertensive medications may affect these results. The beneficial effects of anti-hypertensive medications that target the renin-angiotensin-aldosterone system (RAAS) in DR and DME have been evaluated in several clinical trials, such as the Diabetic Retinopathy Candesartan Trials (DIRECT) and Renin-Angiotensin System Study (RASS).

A recent meta-analysis revealed that patients with DME or PDR were more likely to have incident cardiovascular disease (CVD) and fatal CVD when compared to those without DME or PDR in type 2 diabetes mellitus [44]. It is accepted that fluid retention due to cardiac failure, or another CVD can exacerbate DME and may be an important concern when managing it [45]. In the present study, DME was detected in 20.5% (16) of the patients with type 2 diabetes and CVD and in 14.0% (43) of those without CVD, but the difference was not statistically significant (*p* = 0.151). However, the patients were not examined by a cardiologist, the subclinical findings may not have been noticed, and/or the patients may not have been aware of their CVD.

The prevalence of DME was significantly higher in those patients who consumed alcohol (50%) (*p* = 0.010). In the advanced analysis, alcohol consumption was seen to increase the odds-relative risk 5.95-fold (95% CI: 1.67 < OR < 21.19). This could be due to the deleterious effects of alcohol on glycemic control or because of the compromised treatment compliance in patients who drink regularly. However, in a previous study from Turkey, there was no significant correlation between alcohol consumption and the prevalence of DME [33].

Dyslipidemia has been implicated as an independent risk factor for vision loss and DME [46–48]; however, no single lipid measure has been found to be consistently associated with DR or DME [49]. Of the recent studies, only the Madrid Diabetes Study determined an association between low density lipoprotein cholesterol (LDL-C) and DR incidence [50]. In the current study, the HDL-C was significantly lower in the patients with DME (*p* = 0.046). Moreover, the prevalences of DME in the patients who were and were not using antihyperlipidemic drugs were 9.2% and 17.4%, respectively (*p* = 0.040). Of the 109 patients using antihyperlipidemic drugs, 106 were taking statins. In a 2004 study, it was reported that the atorvastatin in statins reduced the severity of hard exudates and the migration of subfoveal lipids in CSME in dyslipidemic type 2 diabetic patients [51]. In another study from Greece, the use of atorvastatin reduced the severity of hard exudates and fluorescein leakage in diabetic maculopathy in dyslipidemic diabetic patients [52]. In DME-associated lipid exudates, there will generally be a spontaneous resolution over 2 years or longer [53]. Macrophages clear the exudates by phagocytosis [54], and the clearance of lipid exudates in DME can be independently accelerated by serum lipid control and by focal/grid photocoagulation [51]. With decreasing serum lipid levels, statins are also thought to reduce inflammation and secondary microvascular leukocytosis [54]. In contrast, one meta-analysis reported the dose-dependent relationship between statin use and an increased risk of diabetes [55]. This led to the belief that statins might influence glucose homeostasis by decreasing insulin production or increasing insulin resistance, or both [56]. Consequently, the effects of statins on diabetes and DME remain controversial.

The mean serum creatinine levels in patients with and without DME were 1.13 ± 0.81 mg/dl and 0.87 ± 0.63 mg/dl, respectively, and this difference was statistically significant (*p* = 0.021). While 11% of the patients without nephropathy had DME, 29.0% of the patients with microalbuminuria and 26.7% of patients with macroalbuminuria did (*p* < 0.001). In a 15-year follow-up study, the development of macroalbuminuria was found to be associated with the development of DME in type 1 diabetes [57]. In this study, not only macroalbuminuria, but also microalbuminuria was associated with DME.

The major ocular risk factor associated with DME is DR severity. Although DME can be seen at any level of DR, an increasing DR severity has been associated with an increasing prevalence of DME [58–62]. In one study, the 14-year incidence of DME increased from 25% to 37% as the baseline retinopathy severity increased from mild to moderate NPDR [60]. In addition, point estimates of 4% and 15% for the prevalence of subclinical DME in mild to moderate NPDR and severe NPDR to PDR, respectively, have been reported. In this study, the DME prevalence rate was 28.6% in those patients with mild-moderate NPDR, while it was 72.6% in those patients with severe NPDR to PDR (*p* < 0.001).

Starling’s law explains the balance between intravascular and extravascular liquid passage. Based on this, a study published decades ago advocated the idea that high IOP levels protect against the development of exudates [62]. However, there has not yet been enough research done in this regard. In this study, the mean IOPs in those eyes with and without DME were 14.91 ± 2.45 mmHg and 15.12 ± 2.64 mmHg, respectively, but the difference was not statistically significant (*p* = 0.562). Although the findings are inconsistent, diabetes has been found to be a risk factor for developing primary glaucoma in some population-based studies [63]. For instance, the Singapore Malay Eye Study found an association between ocular hypertension and diabetes, but not glaucoma [64].

Diabetes is associated with the early and rapid development of cataracts, and cataract surgery, other types of intraocular surgery, and ocular inflammatory disease may produce inflammatory and angiogenic mediators that can produce macular edema in eyes with or without DR [65–69]. In accordance with this, in the present study, the DME prevalences in the phakic and pseudophakic eyes were 12.9% (49) and 43.7% (14), respectively (*p* < 0.001).

## Conclusions

In 2010, the prevalence of diabetes in Turkey was 13.7% as reported in the Turkish Diabetes Epidemiology II (TURDEP-II) study. In the USA, DR is the leading cause of blindness in individuals aged < 60 years old, and DME is the most common cause of visual loss in those with DR [56, 66]. Fortunately, permanent vision loss can be prevented by the early diagnosis and treatment of DME. The DME prevalence has been reported at a wide range of rates in numerous studies in the literature, but there have been no previous studies in Turkey on this topic. The development of DME may be avoided or limited and the response to treatment may be improved by the regulation of the DME risk factors. In this study, the prevalence of DME was associated with male gender, diabetes duration, HbA1c ≥ 7.0%, insulin usage, alcohol consumption, low HDL-C levels, nephropathy, neuropathy, severity of DR, and previous cataract surgery. However, antihyperlipidemic drugs may be protective against DME. The cross-sectional design could be considered a limitation of this study; therefore, longitudinal studies with more subjects are needed.

## Abbreviations

BCVA: Best corrected visual acuity
CMT: Central macular thickness
CSME: Clinically significant macular edema
DCCT: Diabetes Control and Complications Trial
DIRECT: Diabetic Retinopathy Candesartan Trials
DME: Diabetic macular edema
DR: Diabetic retinopathy
DRCR.net: Diabetic Retinopathy Clinical Research network
EDIC: Epidemiology of Diabetes Interventions and Complications
HbA1c: Hemoglobin A1c
HDL-C: High density lipoprotein cholesterol
IOP: Intraocular pressure
LDL-C: Low density lipoprotein cholesterol
NPDR: Non-proliferative diabetic retinopathy
OCT: Optical coherence tomography
PDR: Proliferative diabetic retinopathy
RAAS: Renin-angiotensin-aldosterone system
RASS: Renin-Angiotensin System Study
VEGF: Vascular endothelial growth factor
